# Maintenance Costs and Advanced Maintenance Techniques in Manufacturing Machinery: Survey and Analysis

**DOI:** 10.36001/ijphm.2021.v12i1.2883

**Published:** 2021

**Authors:** Douglas Thomas, Brian Weiss

**Affiliations:** 1,2 National Institute of Standards and Technology, Gaithersburg, MD, 20899, USA

## Abstract

The costs/benefits associated with investing in advanced maintenance techniques is not well understood. Using data collected from manufacturers, we estimate the national losses due to inadequate maintenance and make comparisons between those that rely on reactive maintenance, preventive maintenance, and predictive maintenance. The total annual costs/losses associated with maintenance is estimated to be on average $222.0 billion, as estimated using Monte Carlo analysis. Respondents were categorized into three groups and compared. The first group is the top 50 % of respondents that rely on reactive maintenance, measured in expenditures. The remaining respondents were split in half based on their reliance on predictive maintenance. The top 50 % of respondents in using reactive maintenance, measured in expenditures, compared to the other respondents suggests that there are substantial benefits of moving away from reactive maintenance toward preventive and/or predictive maintenance. The bottom 50 %, which relies more heavily on predictive and preventive maintenance, had 52.7 % less unplanned downtime and 78.5 % less defects. The comparison between the smaller two groups, which rely more heavily on preventive and predictive maintenance, shows that there is 18.5 % less unplanned downtime and 87.3 % less defects for those that rely more on predictive than preventive.

## 1. INTRODUCTION

The manufacturing industry has evolved considerably at the onset of the 21st century due to the emergence of advanced technologies (Kumar 2018) in the domains of machine tools, robotics, and additive manufacturing. Coupled with the digital connectivity, cybersecurity, and cutting-edge analytics of Smart Manufacturing (Helu 2015) (sometimes referred to as Industry 4.0 [Kolberg and Zuhlke 2015]), manufacturers are deploying more complex processes and techniques on their factory floors to increase their competitiveness in the global marketplace. Although these advanced technologies have promoted enhanced maintenance practices within the factory, the added complexity of Smart Manufacturing technologies has led to new faults and failures. In 2016, maintenance expenditures and preventable losses in discrete manufacturing were estimated in the $193.6 billion U.S. dollars (Thomas and Weiss 2020).

Many in the manufacturing community have turned their attention to advancing monitoring, diagnostic, and prognostic technologies to enhance maintenance strategies (Jin et al. 2016a; Pellegrino et al. 2016). Likewise, end-users of these maintenance-driven technologies represent a diverse group of manufacturers from small to large enterprises (Jin et al. 2016b; Helu and Weiss 2016). Manufacturers typically rely on one or more approaches to maintenance:

- Reactive Maintenance – maintenance is not performed until a process or piece of equipment fails or performs below its necessary specification(s).
- Preventive Maintenance – specific maintenance activities are scheduled based upon expected units of time or/and cycles
- Predictive Maintenance – specific maintenance activities are orchestrated based upon the monitoring of measures or metrics that would indicate a decreased health (or performance) condition of a process or piece of equipment

Before an organization chooses to invest, it is advantageous for them to estimate the expected return on investment of advancing their maintenance capabilities. This can present a daunting task for manufacturers, particularly small enterprises. Creating a reasonable estimate relies upon numerous factors including 1) understanding one’s current maintenance practices, including existing technologies and their resultant maintenance costs (or savings), 2) identifying the most appropriate technologies that could be viable candidates for integration into the existing manufacturing ecosystem, and 3) the resultant initial expenditures and ultimate cost savings, ideally, of integrating a new technology into the existing manufacturing environment.

This article presents the findings and corresponding analysis of a survey instrument that was recently deployed to maintenance managers in selected manufacturing industries. We estimate the national losses due to inadequate maintenance. We then make comparisons between those that rely on reactive maintenance, preventive maintenance, and predictive maintenance. The results demonstrate potential savings at both the national level and establishment levels.

## 2. LITERATURE

We identify five major aspects in the economics of advanced maintenance:

1. Maintenance costs
2. Benefits of advanced maintenance techniques
3. Utilization of different maintenance techniques
4. Barriers to adopting advanced maintenance techniques
5. Methods for conducting an investment analysis of advanced maintenance

In a previously published report, we discuss at length the literature on the first four items (Thomas and Weiss 2018). This paper, however, is relevant only to items one through three at the aggregated industry level. Literature on these topics are discussed below. This paper does not conduct an investment analysis (i.e., item five); rather, it estimates costs and benefits at the industry level. For more information on investment analysis, Thomas (2017) presents methods for conducting such an assessment and NIST’s Smart Investment Tool (Thomas 2020) implements them. There also exists other literature that discusses investment issues in a more specific context related to maintenance (e.g., Hou-bo et al. 2011; Feldman et al. 2008; and Hölzel 2015).

### Maintenance Costs:

The estimates for manufacturing machinery maintenance costs have a wide range from 15 % to 70 % of the cost of goods produced (Thomas and Weiss 2018), as illustrated in Table 1. The literature, typically, uses varying metrics and range in their estimates, as shown in the table. For instance, Komonen estimates that industrial maintenance is 5.5 % of company turnover (i.e., sales) but it ranges from 0.5 % to 25 % (Komonen 2002). Other research estimates that maintenance is 37.5 % of the total cost of ownership (Herrmann et al. 2011). Along with the variation in metrics, the literature on maintenance costs varies by country and industry of study, making it difficult to compare and generalize.

### Benefits of Advanced Maintenance Techniques:

There are a number of maintenance strategy terms, including maintenance prevention, reliability centered maintenance, computerized maintenance, total predictive maintenance, productive maintenance, and total productive maintenance.

**Figure 1.**
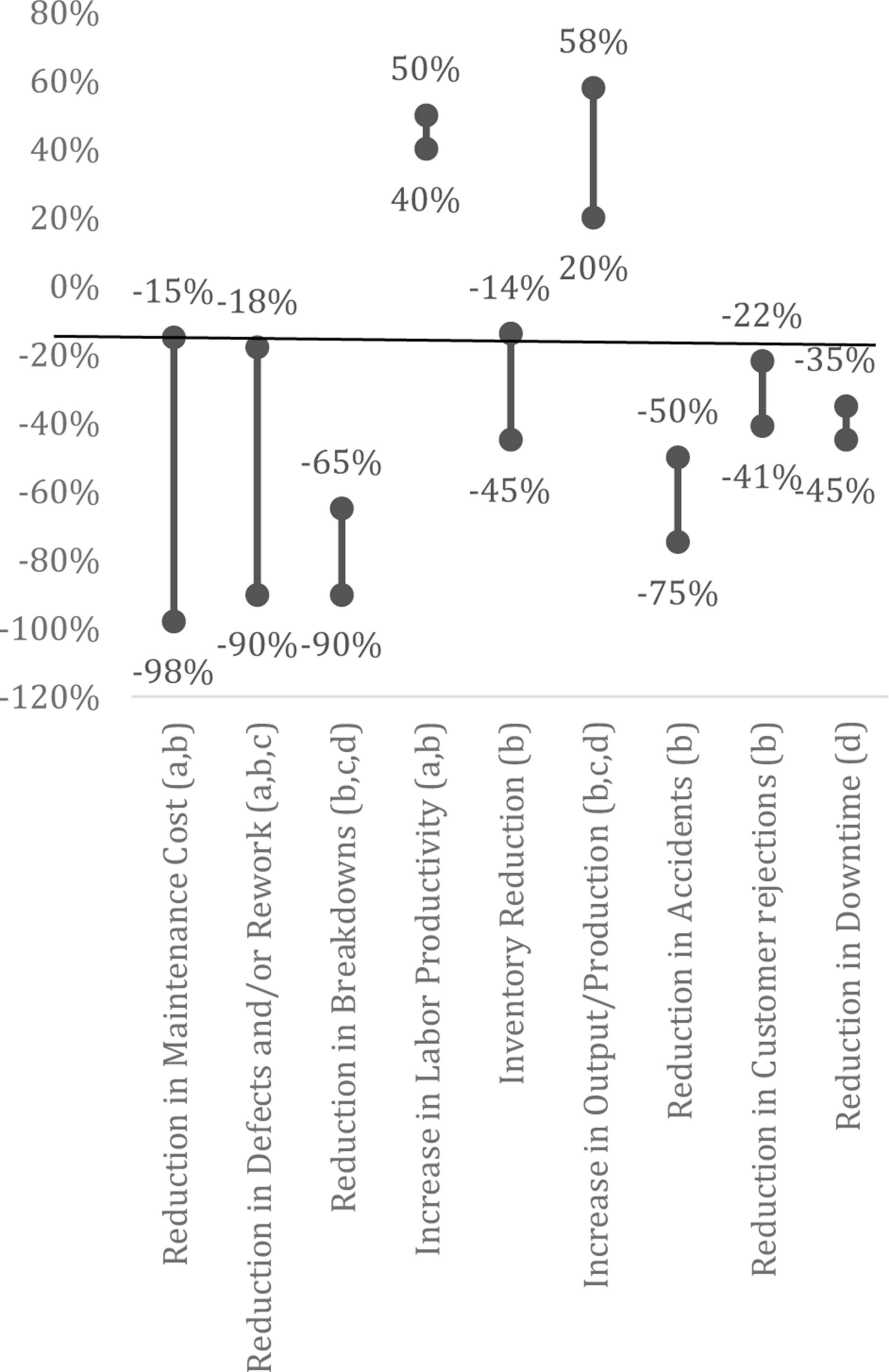
Range of Impacts Identified in Various Publications for Implementing Advanced Maintenance Techniques, Percent Change Sources: ^a^Nakajima, S. Introduction to Total Productive Maintenance (TPM). (Portland, OR: Productivity Press, 1988). ^b^Ahuja, I.P.S. and J.S. Khamba. “Total Productive Maintenance: Literature Review and Directions.” International Journal of Quality and Reliability Management. 25, no 7 (2008): 709–756. ^c^Chowdhury, C. “NITIE and HINDALCO give a new dimension to TPM.” Udyog Pragati, Vol. 22 No. 1, (1995): 5–11. ^d^Federal Energy Management Program. Operations and Maintenance Best Practices: A Guide to Achieving Operational Efficiency. (2010). https://energy.gov/sites/prod/files/2013/10/f3/omguide_complete.pdf

Some of the terms are not used consistently in the literature (Thomas and Weiss 2018). Advanced maintenance techniques have been shown to have a range of impacts, which have been measured using varying metrics, as shown in Figure 1.

### Utilization of Different Maintenance Techniques:

A study by Helu and Weiss (2016) suggests, using anecdotal evidence, that small and medium firms might rely more heavily on reactive maintenance with limited amounts of predictive maintenance. Meanwhile, larger firms seem to rely on preventive maintenance. A survey of Swedish firms (Alsyouf 2009) suggests that 50 % of maintenance time is spent on planned tasks with 37 % on unplanned tasks and 13 % for planning. Approximately 70 % considered maintenance a cost rather than an investment or source of profit.

**Figure 2.**
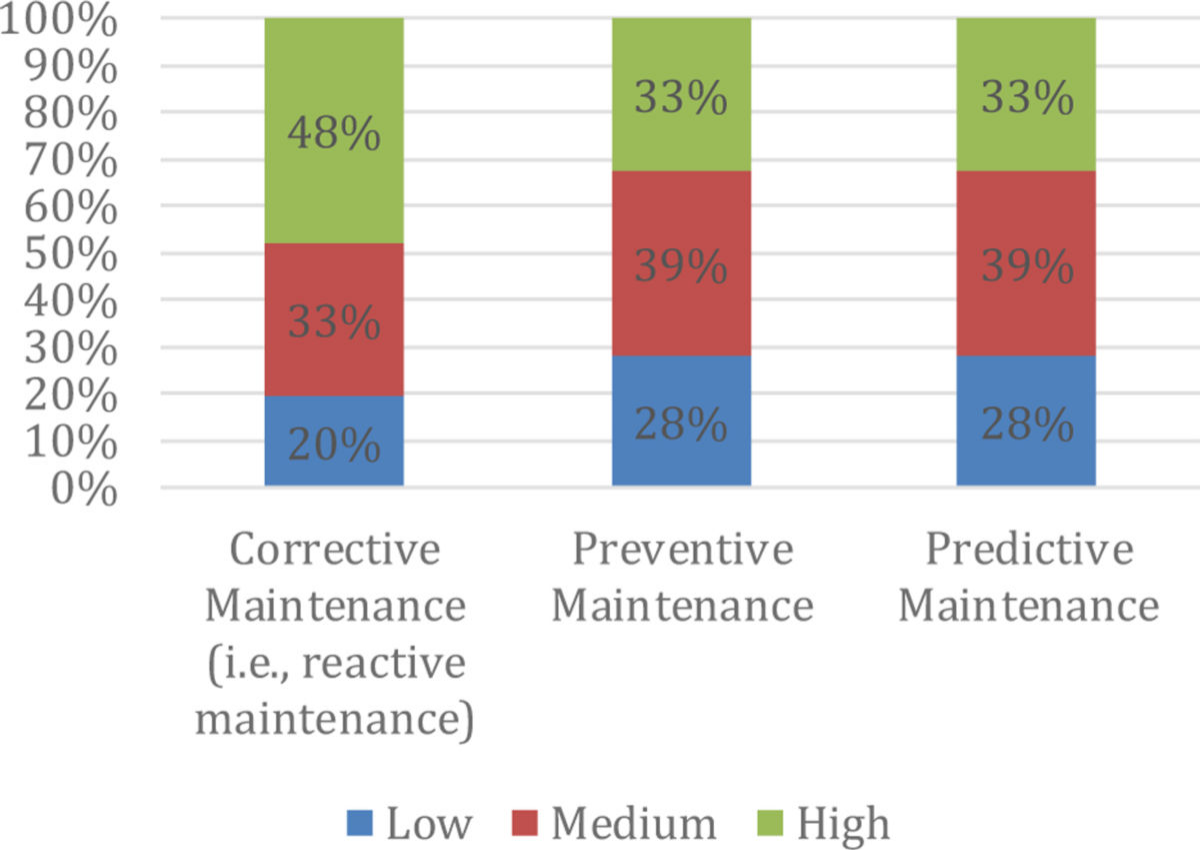
Maintenance by Type (Percent of Respondents out of a Total of 46) Source: Pinjala, Srinivas Kumar, Liliane Pintelon, and Ann Vereecke. An Empirical Investigation on the Relationship between Business and Maintenance Strategies.” International Journal of Production Economics. 104. (2006): 214–229.

Another survey, from Belgium, provides the number of respondents that indicated that they have a high, medium, or low level of each of the different maintenance types, as seen in Figure 2. Approximately 48% indicated that they rely on reactive maintenance to a high level. Another survey (Jin et al. 2016a; Jin et al. 2016b) found that companies are beginning to consider predictive maintenance techniques with a majority of respondents having active projects in manufacturing diagnostics and prognostics. Approximately a quarter of the respondents indicated that they primarily relied on reactive maintenance, though.

Moreover, the literature related to maintenance tends to focus on technological issues. To some extent there are studies that integrate economic data; however, these represent a small amount of the literature (Grubic 2009). Much of the economic literature is individual case studies, personal insights, or other anecdotal observations. Some papers refer to common economic methods that are used in investment analysis. Other papers discuss methods for examining maintenance costs, often with a focus on the technological aspects. Unfortunately, many do not provide data or real examples. There is a gap in the literature where the potential benefits of adoption of predictive maintenance is not well understood at the industry level. This paper measures, at the industry level, maintenance costs and losses that could be avoided with investments in advanced maintenance techniques. It also compares those establishments that rely on reactive maintenance with those that have invested in more advanced maintenance techniques.

## 3. DATA

A survey instrument, the Machinery Maintenance Survey, was used to collect data from manufacturers. The survey targeted managers of machinery maintenance and was distributed through multiple means: mail, email, newsletters, and in-person presentations. We received 85 responses; however, some were removed due to issues with the responses, leaving 71 respondents. For example, some respondents did not complete key questions while others were outside of the manufacturing industry. The survey included questions presented in Appendix A of NIST Advanced Manufacturing Series 100–34 (Thomas and Weiss 2020). The survey was reviewed by numerous practitioners to ensure that the questions were practical.

Surveys sent in the mail were stratified by establishment type to increase the probability that there were responses from different groups of establishments. The groupings were by industry (identified by the North American Industry Classification System or NAICS as codes 333 for machinery, 334 for computers, 335 for electronics, and 336 for transportation equipment) and region. Establishments were selected randomly from three regions (north, south, and west) of the country for a total of 12 stratifications. The survey was anonymous. Table 2 presents the responses by industry by establishment size after removing unused responses.

Two other data sets are utilized for scaling. The first is the 2012 Economic Census (Census Bureau 2020a) that provides data on shipments by industry, using NAICS codes, and by establishment size. Note that the term “shipments” is defined by the Census Bureau as the value of products sold. The second data set is the Annual Survey of Manufactures (ASM), which provides data by NAICS code (Census Bureau 2020b).

## 4. METHODS

In order to quantify the uncertainty in scaling the survey results, this paper utilizes a combination of Monte Carlo analysis and resampling. It expands on work published in NIST Advanced Manufacturing Series 100–34 (Thomas and Weiss 2020). Monte Carlo analysis is based on works by McKay, Conover, and Beckman (Mckay et al. 1979) and by Harris (1984) that involves a method of model sampling. It can be implemented using a software package such as the Monte Carlo Tool provided by NIST (National Institute of Standards and Technology 2019), which was used for this analysis. Specification includes selecting variables to be simulated, the distribution of the variables, and the number of iterations performed. A software tool then randomly samples from the probabilities for each input variable. Common distributions include triangular, normal, and uniform distribution. In this instance, the analysis has 10 000 iterations. The second part of the method includes resampling where repeated samples are taken from another sample.

In the Monte Carlo analysis one of five methods for scaling data, described below, is selected randomly for each iteration. Each method has between one and four stratifications described below. The resampling part of the method involves resampling from each of these stratifications for each Monte Carlo iteration. For resampling, 75 % of the responses are selected randomly and calculations are made from them. This level was selected as it allows for any potential outliers to be excluded in some of the Monte Carlo iterations, but keeps the number selected reasonably high. Additionally, some values are adjusted using the producer price index (PPI). In the Monte Carlo analysis, the PPI is varied by plus/minus 20 % using a triangular distribution with the most likely value being the observed value. This level was selected to allow for significant variation in the adjustment for inflation.

Six maintenance related items are estimated:

- Item 1: Direct maintenance costs (e.g., maintenance department)
- Item 2: Additional maintenance costs due to faults and failures
- Item 3: Inventory costs for finished goods associated with maintenance
- Item 4: Unplanned downtime costs associated with maintenance
- Item 5: Lost sales due to maintenance issues
- Item 6: Defects due to maintenance issues

For these estimates, the responses are scaled-up to represent national level estimates using industry data on shipments. Additionally, the data is stratified by industry and/or employment size. We use three different stratifications, as outlined in Table 3. Stratification is used to address any over/under representation of groups that might skew estimates. Manufacturers might have varying maintenance costs as a result of the types of products they produce or the size of their establishment. If a group is not represented accurately, the aggregate estimate can be skewed. The first strata is a blend of industry and establishment size, as shown in Table 3. In this strata, industry *i* alternates between two sets of industries: NAICS 321–333, 337 and NAICS 334–336, 339. The establishment size *s* alternates between “1 to 99 employees” and “100 or more employees.”

As shown in Table 3, the second strata is by employment alone. In this strata, the industry is constant (i.e., NAICS 321–339 excluding 324 and 325) with establishment size varying between the three groups. The third strata is by industry, as seen in Table 3. In this strata, establishment size is constant (i.e., all sizes) while industry i varies between the three groups. Two estimates are made using this strata. The strata are used within five methods for estimating maintenance cost/loss items. Each method is scaled using either Economic Census data or data from the Annual Survey of Manufactures.

They also either use one of the three stratifications or have no stratification:

- Method 1: Stratification by strata 1 and scaled using Economic Census data
- Method 2: Stratification by strata 2 and scaled using Economic Census data
- Method 3: Stratification by strata 3 and scaled using Economic Census data
- Method 4: Stratification by strata 3 and scaled using Annual Survey of Manufactures data
- Method 5: No stratification and scaled using Annual Survey of Manufactures data

The stratification groups were selected to keep a minimum number of establishments in each group so that there is enough respondents representing that group. The Monte Carlo analysis selects randomly from the five scaling methods. The calculation for each maintenance cost/loss item for each method of estimation can be represented as the following:

$$MC_{m,i}=\sum_{g=1}^{G}\left(\frac{\sum_{x=1}^{X}EM_{x,m,g,i}}{\sum_{x=1}^{X}SM_{x,m,g}}SM_{TOT,m,g}\right)$$

where

$MC_{m,i}$= Maintenance cost item *i* estimated using method *m*, where *i* is one of the six maintenance items discussed above and *m* is one of the five methods for estimation discussed below

$EM_{x,m,g,i}$= Estimate of maintenance costs/loss item *i* for establishment *x* in group *g* of method *m*, where *i* is one of the six maintenance items discussed above, *m* is one of the five methods discussed above, and *g* is either one of the groups within each method (see Table 3) or the total of all establishments if no strata is used.

*SM*_*x,m,g*_ = The scaling metric, shipments, from establishment *x* in group *g* of method *m*, where *m* is one of the five methods discussed above and *g* is either one of the groups within each method (see Table 3) or the total of all establishments if no strata is used.

*SM*_*TOT,m,g*_ = Total shipments from either the Annual Survey of Manufactures or Economic Census (depending on the method used) for group *g* of method *m*, where *m* is one of the five methods discussed above and *g* is either one of the groups within each method (see Table 3) or the total of all establishments if no strata is used

Moreover, each iteration of the Monte Carlo analysis estimates six maintenance cost/loss items using one of five estimation methods selected randomly, which have varying stratifications that include between 1 and 4 groups. Approximately 75 % of the respondents in a group are selected randomly to estimate the maintenance cost/loss items. The producer price index, used to adjust some dollar values, is varied by plus/minus 20 % in a triangular distribution.

Finally, for two comparisons, seven additional metrics are estimated from each iteration of the Monte Carlo analysis:

- Metric 1: Average ratio of maintenance costs to total shipments
- Metric 2: Additional maintenance costs due to faults and failures as a ratio to shipments
- Metric 3: Percent of planned production time that is downtime
- Metric 4: Defect rate
- Metric 5: Percent of sales lost due to delays related to maintenance
- Metric 6: Percent of sales lost due to defects related to maintenance
- Metric 7: Percent increase in inventory due to maintenance issues

**Figure 3.**
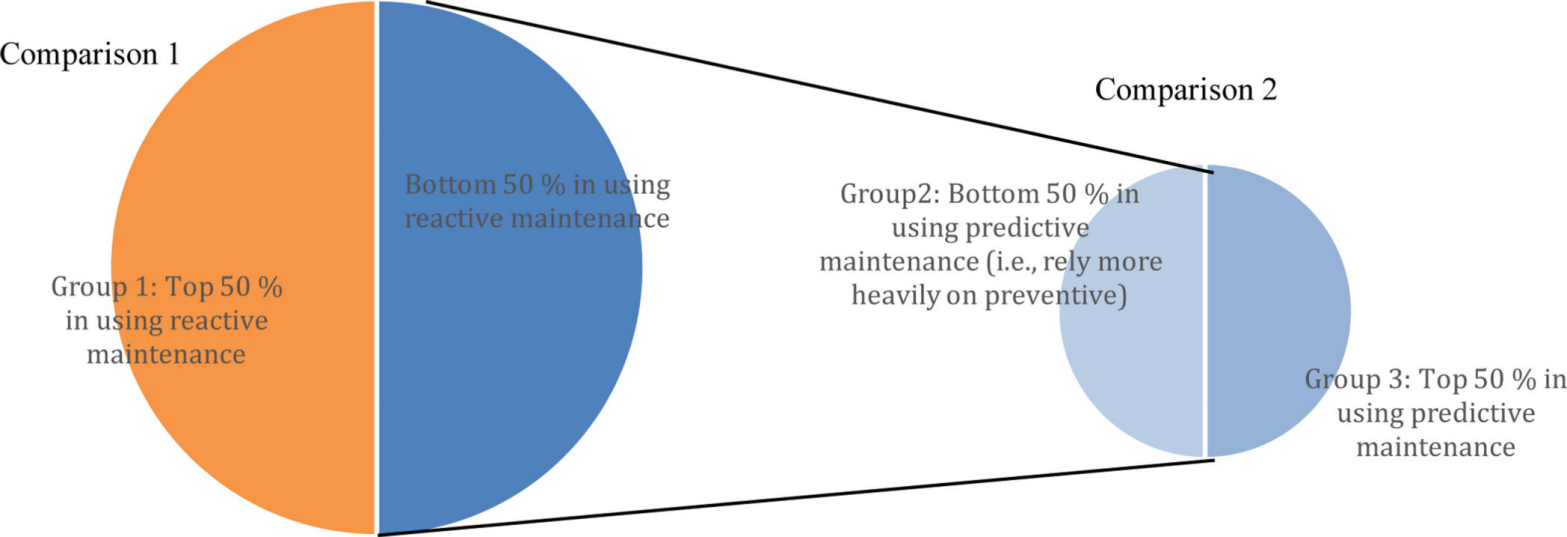
Illustration of the Comparison for each Monte Carlo Iteration

Respondents were categorized into three groups based on the types of maintenance that they use. The first group is the top 50 % of respondents that rely on reactive maintenance, measured in maintenance expenditures. The remaining respondents were split in half based on their reliance on predictive maintenance: the half relying more on predictive and the half that rely more on preventive. Two comparisons are made with the first being between those in the top 50 % in using reactive maintenance and the other two groups. The second is between the two smaller groups relying on predictive and preventive maintenance. The two comparisons are illustrated in Figure 3. The average for each metric within the groups is compared. Since these are not dollar values, they are not scaled. Moreover, the estimates for the comparisons are not affected by the selection of the method, group, or variation in the PPI as they are not used to calculate any of the seven metrics.

**Figure 4:**
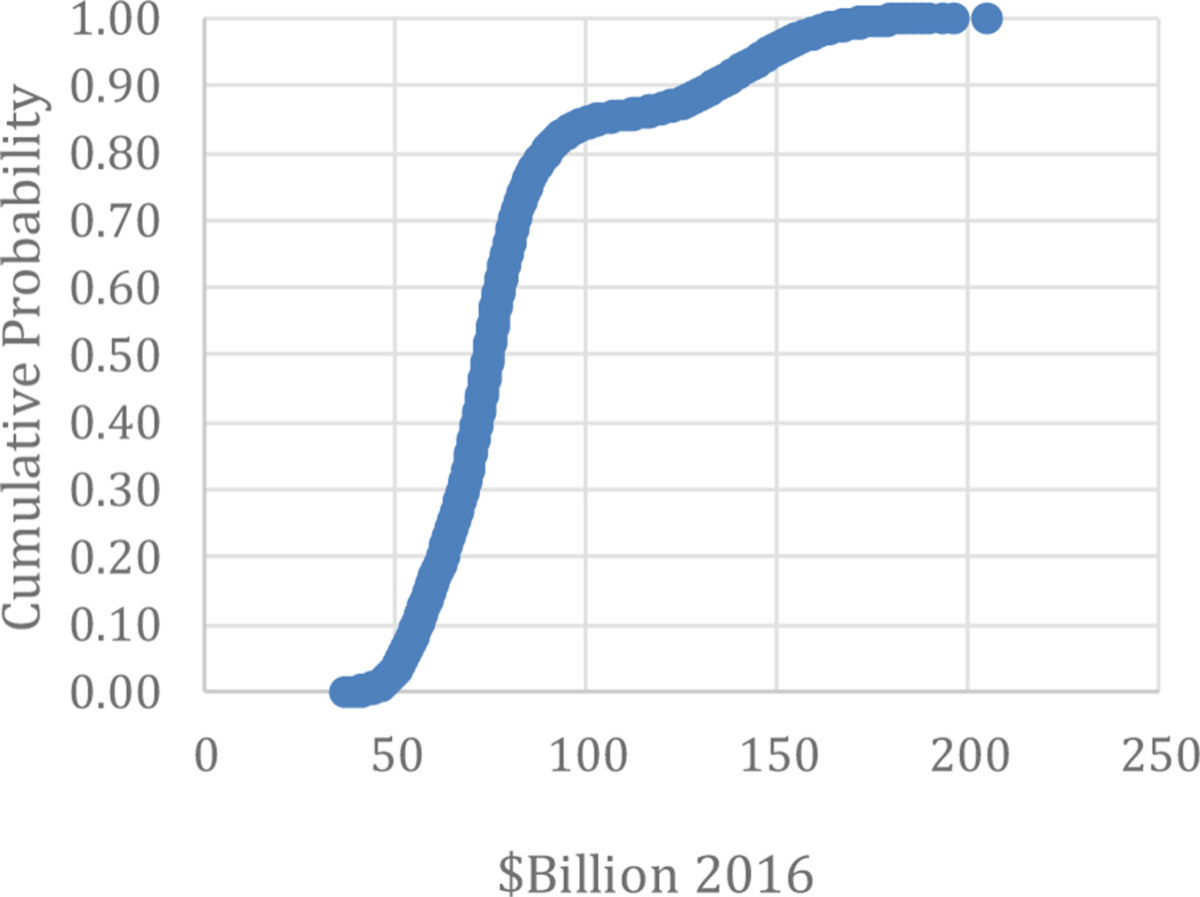
Monte Carlo Results: Direct Maintenance Costs

Comparing these items can provide insight into the benefits of adopting advanced maintenance for a manufacturer. These two comparisons seem to be the most relevant for understanding the costs and benefits of investing in different maintenance options. The seven metrics are the primary factors collected from manufacturers to estimate the costs and losses associated with maintenance and can be used by Direct Maintenance Costs: As seen in Table 4, direct maintenance costs are estimated to be $57.3 billion for the U.S., estimated by Thomas and Weiss (2020) using no stratification and no Monte Carlo analysis. The 90 % confidence interval for this estimate ranges from $50.8 billion to $103.3 billion. The results of the Monte Carlo analysis in this paper puts the estimate a bit higher with the average of the simulation output being $81.6 billion and the median being $74.4 billion (see Table 5). The range is between $36.7 billion and $205.4 billion, as illustrated in Figure 4. Although the range extends to over $200 billion, 85 % of the values are below $102.9 billion. Moreover, the true value of Direct Maintenance Costs is likely at or below this amount.

**Figure 5.**
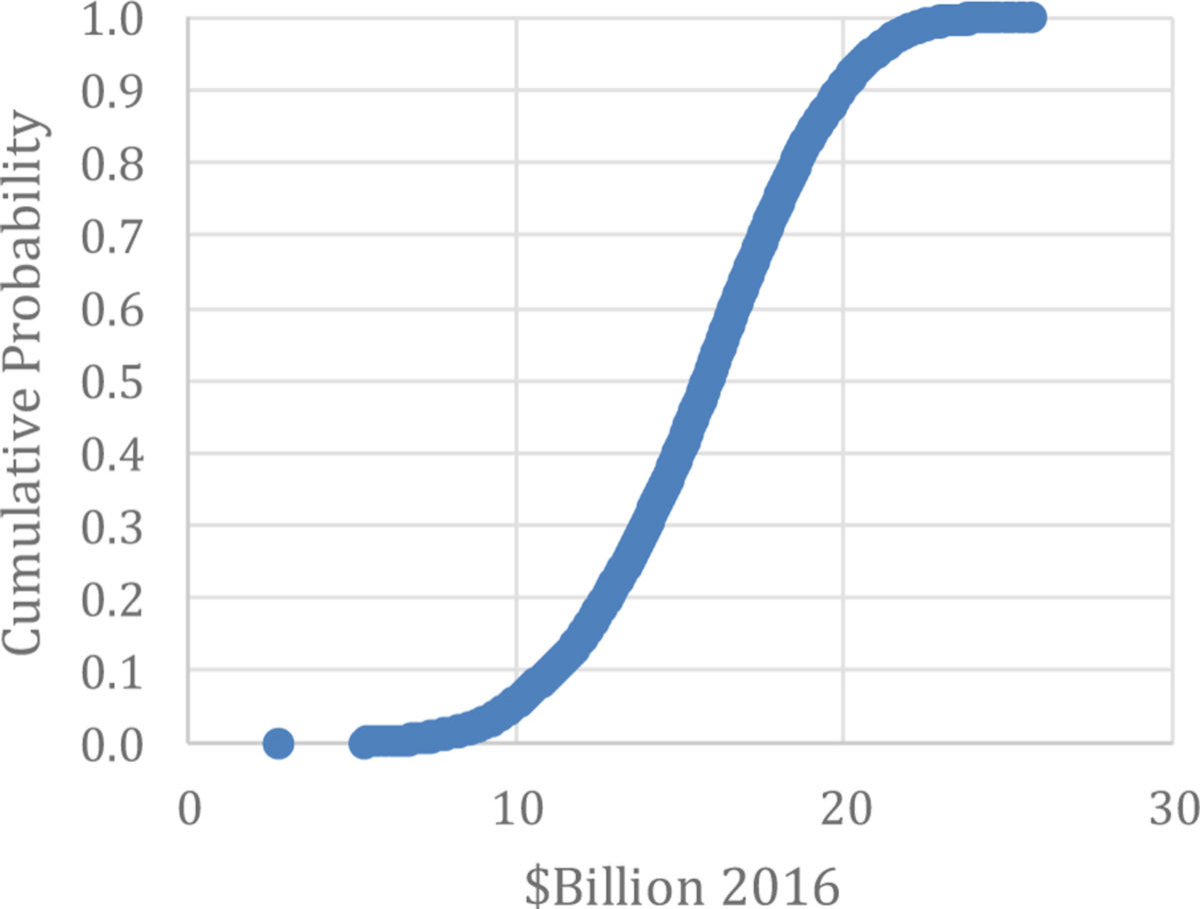
Monte Carlo Results: Additional Costs due to Faults and Failures

Costs due to Faults and Failures: Costs due to faults and failures were estimated to be $16.3 billion (estimated by Thomas and Weiss (2020) without stratification) as seen in Table 4. This is not too different than the average and median of the Monte Carlo results in this paper, which are $15.7 billion and $15.8 billion, respectively. The range is between $2.7 billion and $25.7 billion, as illustrated in Figure 5. Approximately 85 % of the values are below $19.2 billion.

**Figure 6.**
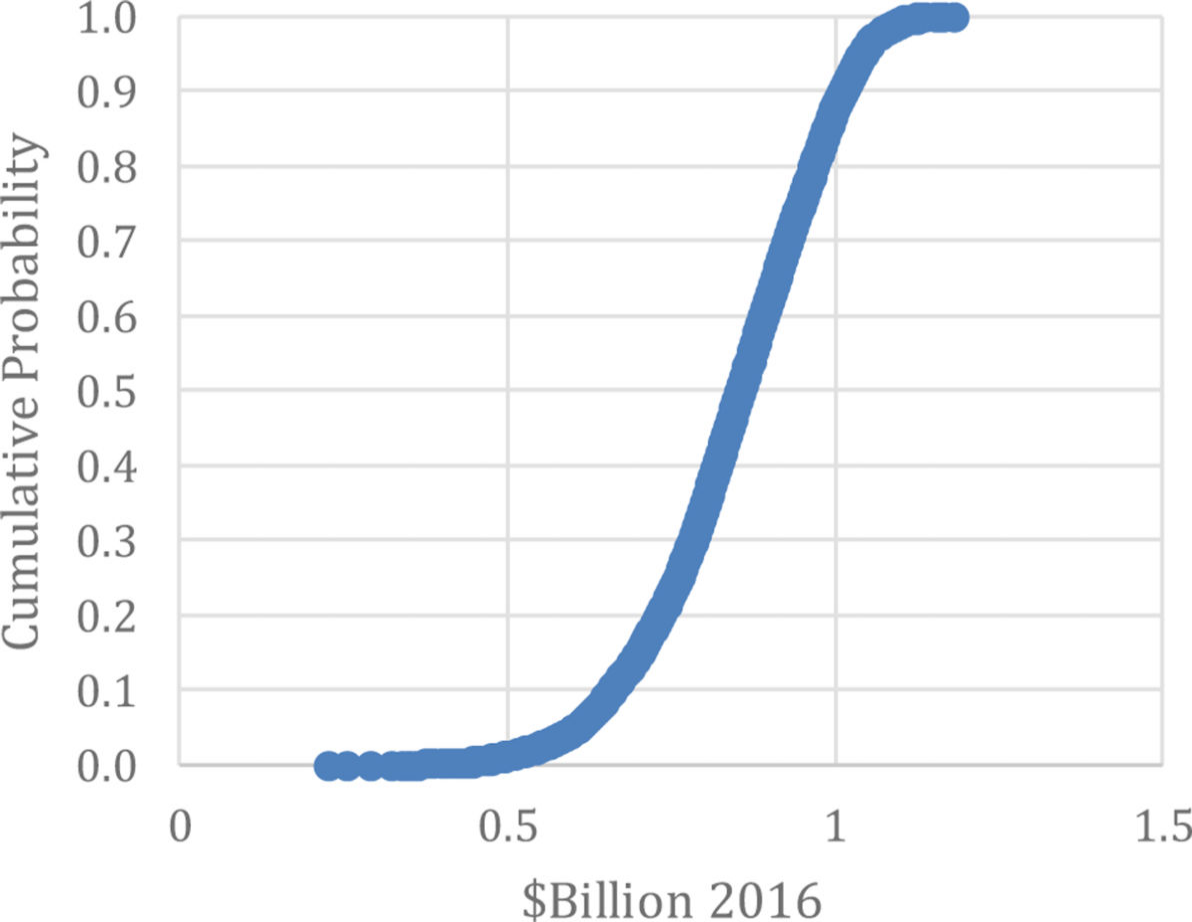
Monte Carlo Results: Inventory Costs

Inventory Costs: Manufacturers maintain finished goods inventory to mitigate the risks of disruptions. Costs for inventory associated with maintenance issues is estimated by Thomas and Weiss (2020) to be $0.9 billion with a 90 % confidence interval between $0.3 billion and $1.1 billion, as calculated without stratification or using a Monte Carlo analysis (see Table 4). The average and median of the Monte Carlo results were $0.8 billion and $0.9 billion (see Table 5), which is not much different than that of the unstratified estimate. The range of the iterations were between $0.2 and $1.2 billion, as illustrated in Figure 6.

which is not much different than that of the unstratified estimate. The range of the iterations were between $0.2 and $1.2 billion, as illustrated in Figure 6.

**Figure 7.**
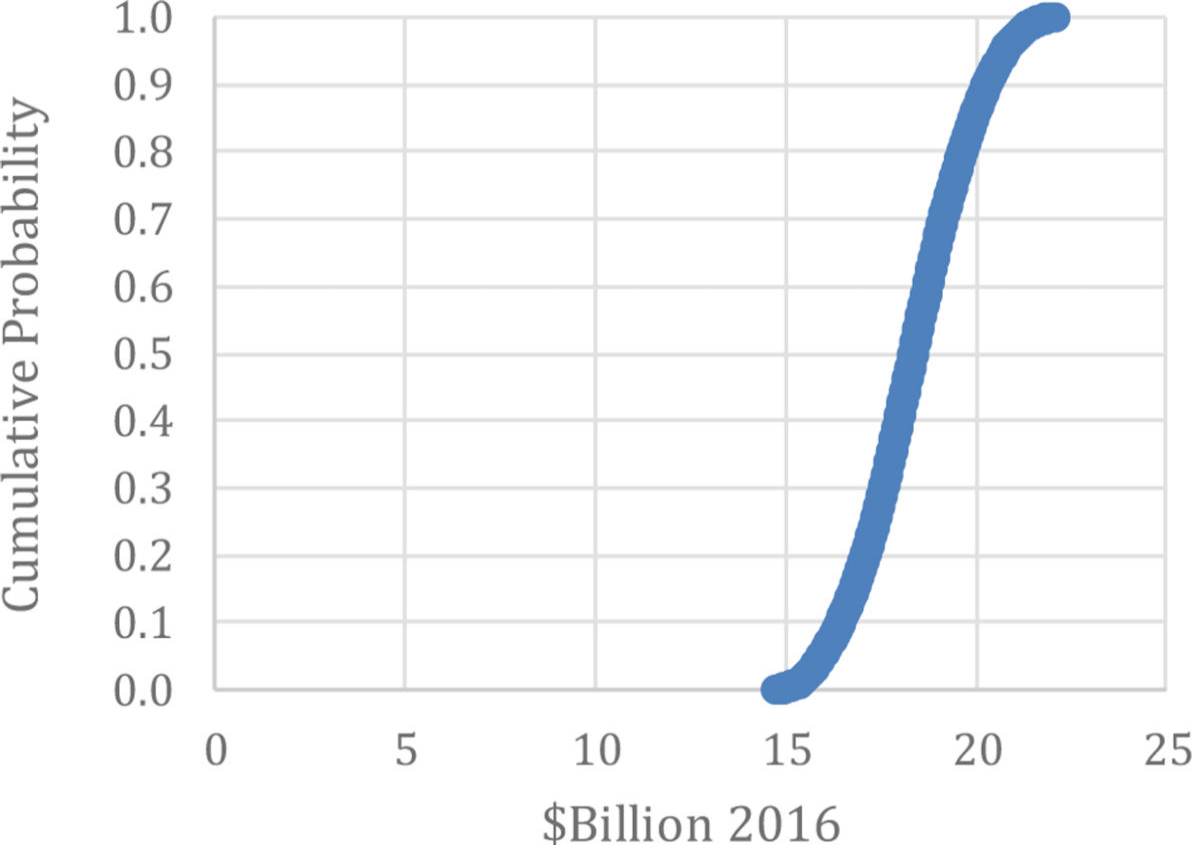
Monte Carlo Results: Downtime Costs

Unplanned Downtime: Unplanned downtime losses were estimated by Thomas and Weiss (2020) to be $18.1 billion with a 90 % confidence interval between $10.4 billion and $27.8 billion, estimated without stratification or Monte Carlo analysis. This is not much different than the average and median from the Monte Carlo analysis in this paper, which are both $18.4 billion. The range for the analysis is between $14.8 billion and $22.1 billion, as seen in Figure 7.

**Figure 8.**
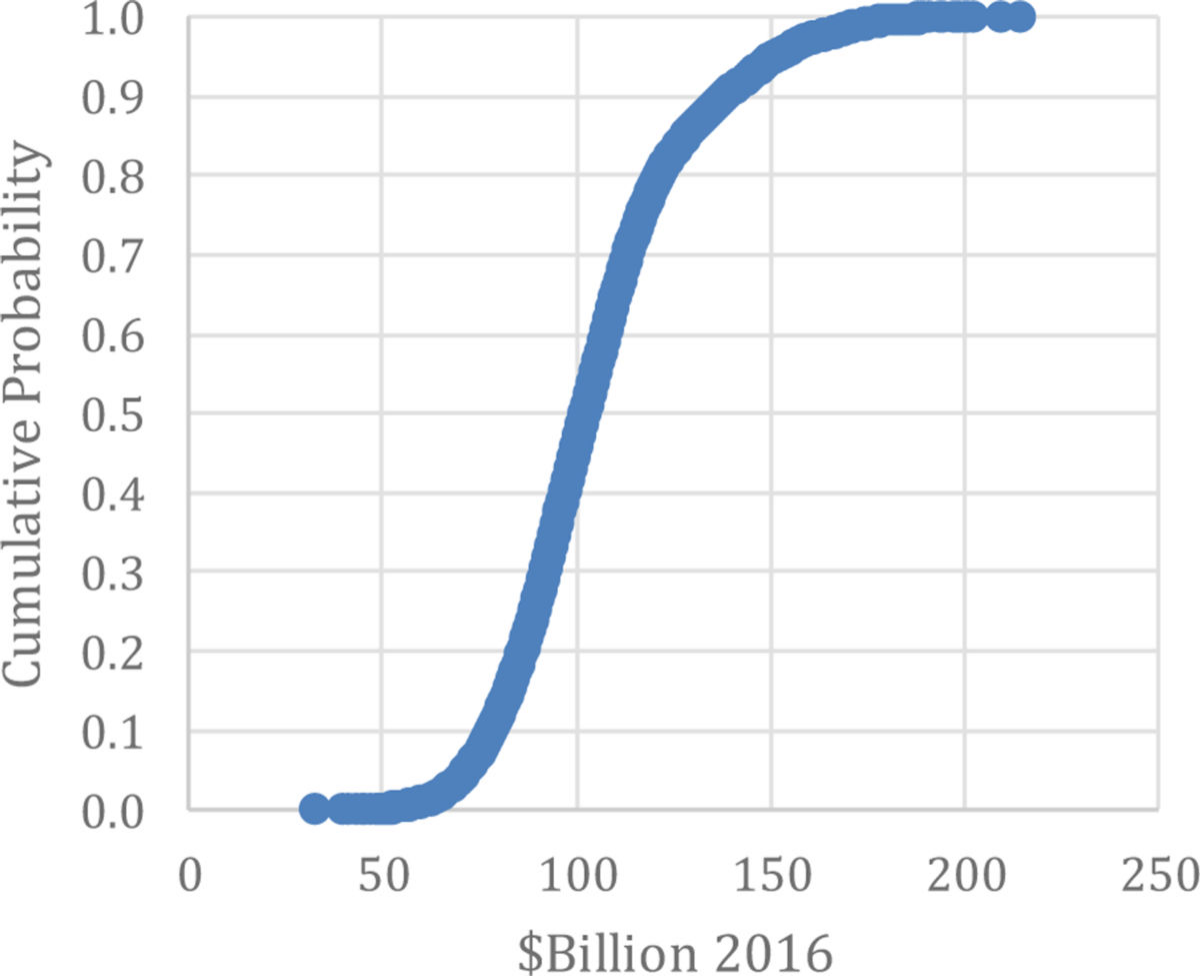
Monte Carlo Results: Lost Sales

Lost Sales: The estimate without stratification for lost sales from Thomas and Weiss (2020) is $100.2 billion with a 90 % confidence interval between $33.5 billion and $166.8 billion. The Monte Carlo analysis has an average and median that is not much different at $105.0 billion and $102.1 billion, respectively. The range is between $32.6 billion and $214.0 billion, as illustrated in Figure 8.

**Figure 9.**
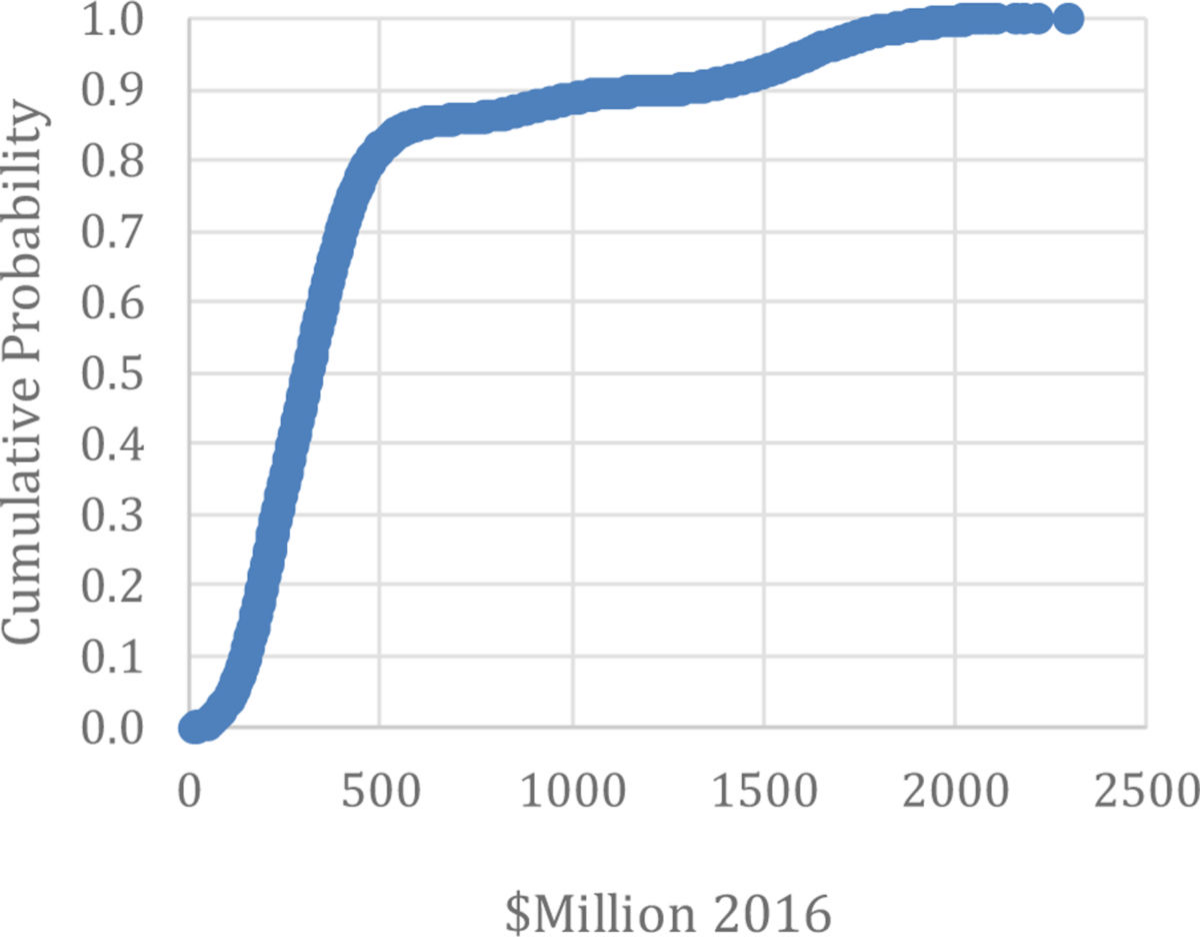
Monte Carlo Results: Defects

Defects due to Maintenance Issues: The estimated losses due to defects associated with maintenance is estimated by Thomas and Weiss (2020) to be $0.8 billion, estimated without stratification or Monte Carlo analysis (see Table 4). The 90 % confidence interval using this method is between $0.0 billion and $2.7 billion. The average and median of the Monte Carlo results are $0.5 billion and $0.3 billion, as seen in Table 5. The range of the results are between less than $0.1 billion and $2.3 billion, as illustrated in Figure 9.

50/50 Comparison – Reactive Maintenance: This analysis compared the top 50 % of establishments using reactive maintenance with the bottom 50 %. This comparison illustrates the costs and benefits of moving away from reactive maintenance toward either predictive or preventive maintenance. The results should be treated as anecdotal evidence, as the groupings result in a smaller number of respondents. Those manufacturers that relied less on reactive maintenance spent on average 81.7 % more on direct maintenance costs relative to their shipments, as shown in Table 6. However, they had 51.8 % less additional costs due to faults/failures in relation to shipments. They also had 52.7 % less unplanned downtime, 78.5 % fewer defects, 49.4 % to 73.0 % less lost sales, and 51.2 % less increase in inventory due to maintenance, as shown in Table 6. On average, those establishments in the top 50 % used reactive maintenance 68.8 % of the time compared to 22.2 % of the time for the bottom 50 %, as seen in Table 5. The implication here is that those manufacturers that invest in either preventive or predictive maintenance may have higher upfront maintenance costs but have fewer losses due to machinery breakdowns or machinery coming out of alignment and producing flawed products. As a result, customers are less likely to be unsatisfied due to product defects or delivery delays. Additionally, the manufacturer will have more reliable production times, as they have fewer delays from unexpected breakdowns.

50/50 Comparison – Predictive Maintenance: An additional comparison was made between those in the top 50 % for using predictive maintenance compared to the bottom 50 %, excluding the top 50 % in using reactive maintenance (see Figure 3 for illustration). That is, we compare those that rely more heavily on preventive and predictive maintenance. Those that rely on reactive maintenance are excluded in order to better isolate the effect of predictive and preventive maintenance. This comparison illustrates the costs and benefits of moving from preventive maintenance toward predictive maintenance. The results should be treated as anecdotal evidence, as the groupings result in a smaller number of respondents. Additional research is needed to confirm these relationships. Direct maintenance costs relative to shipments are 212.3 % higher for the top 50 % using predictive maintenance. This higher cost is, potentially, the cost of investing in predictive maintenance.

Additional costs due to faults and failure are, surprisingly, a little higher. This is unexpected as one would presume that advanced maintenance would reduce faults/failures. This could easily be a result of sample size or other sampling issues in the survey. Another explanation is that there could be a difference in the threshold for identifying a fault or failure. Manufacturers who use more advanced maintenance practices could define fault/failure as a process that is no longer able to maintain necessary quality or productivity targets. Whereas, manufacturers that leverage less advanced maintenance methods could define a fault/failure as an instance where a process or piece of equipment stops working altogether. Under these definitions, those with advanced maintenance strategies would see more faults and failures because they are capturing information more frequently and/or at a finer resolution.

Sales lost were also higher for those relying on predictive maintenance. This might seem unexpected, but it is possible that firms that adopt predictive maintenance have products and customers that are more sensitive to defects and/or delays, which is likely why they adopted predictive maintenance. This seems likely especially since downtime and defects are lower. Downtime is 18.5 % lower, defects are 87.3 % lower, and inventory losses are 22.5 % lower. The implication here is that investing in predictive maintenance provides benefits that exceed that of preventive maintenance, as it further reduces breakdowns and defects.

A third comparison could also be made by comparing the top 50 % in using reactive maintenance (see Table 6), which equates to the orange portion in Figure 3, to the bottom 50 % in using predictive maintenance, which equates to the light blue area of Figure 3 – i.e., those that rely more on preventive maintenance (see Table 7). This provides insight into moving from reactive maintenance to preventive maintenance. Although this was not examined extensively, data between the two tables can be compared. Preventive maintenance has 48.5 % lower unplanned downtime and 63.2 % lower defects.

## 5. CONCLUSION AND SUMMARY

The total costs and losses associated with maintenance is estimated to be on average $222.0 billion with the median being $211.8 billion, as estimated in the Monte Carlo analysis (see Table 5). This estimate is similar to, although slightly higher than, a previous estimate of $194 billion. Approximately half of the costs/losses are due to lost sales. To put these estimates in perspective, the value added for the same industries in 2016 was $1.5 trillion and shipments was $3.2 trillion. These industries spent $491 billion on payroll including maintenance staff, $82 billion on machinery/equipment, $33 billion on electricity, $15 billion on capital expenditures on buildings/structures, and $4 billion on computer hardware/other equipment, according to 2016 data from the Annual Survey of Manufactures (Census Bureau 2020b; Thomas and Weiss 2020). Not only are maintenance costs/losses a major contributor to industry costs, they also amount to a substantial magnitude of money in the U.S. economy.

The comparison of the top 50 % in using reactive maintenance to those in the bottom 50 % suggests that there are substantial benefits of moving away from reactive maintenance toward preventive and/or predictive maintenance. The bottom 50 %, which relies more heavily on predictive and preventive maintenance, had 52.7 % less unplanned downtime and 78.5 % fewer defects. The comparison of the top 50 % in using predictive maintenance compared to the bottom 50 %, excluding those that rely on reactive maintenance, shows that there is 18.5 % less unplanned downtime and 87.3 % less defects for the top 50 %. This suggests there may be significant benefits to adopting predictive maintenance. Individual businesses still need to evaluate investments in maintenance from their current circumstances and competitive strategies. However, this paper provides evidence that there are potentially significant benefits to investing in advanced maintenance. It also provides general guidance as to the types of benefits and magnitude of benefits that might be expected.

## Figures and Tables

**Table 1.** Characteristics of Maintenance Costs from a Selection of Articles, Various Countries/Industries

	Maintenance	
Description	Low	High
Cost of Goods Sold^a,b^	15.0%	70.0%

Sales^c^	0.5%	25.0%

Cost of Ownership^d^	37.5%	

Replacement Value of Plant^e^	1.8%	5.0%

Cost of Manufacturing^f^	23.9%	

Percent of Planned Production Time that is Downtime^f^	13.3%	

Sources:

a Mobley, R. Keith. An Introduction to Predictive Maintenance. (Woburn, MA: Elsevier Science, 2002). 1.

b Bevilacqua, M. and M. Braglia. “The Analytic Hierarchy Process Applied to Maintenance Strategy Selection.” Reliability Engineering and System Safety. 70, no 1 (2000): 71–83.

c Komonen, Kari. “A Cost Model of Industrial Maintenance for Profitability Analysis and Benchmarking.” International Journal of Production Economics. 79 (2002): 15–31.

d Herrmann, C., S. Kara, S. Thiede. “Dynamic Life Cycle Costing Based on Lifetime Prediction.” International Journal of Sustainable Engineering. 4, no 3 (2011): 224–235.

e Eti, M.C., S.O.T. Ogaji, and S.D. Probert. “Reducing the Cost of Preventive Maintenance (PM) through Adopting a Proactive Reliability-Focused Culture.” Applied Energy. 83 (2006): 1235–1248.

f Tabikh, Mohamad. “Downtime Cost and Reduction Analysis: Survey Results.” Master Thesis. KPP321. Mӓlardalen University. (2014). http://www.diva-portal.org/smash/get/diva2:757534/FULLTEXT01.pdf

**Table 2.** Responses to the Survey by Firm Size and NAICS Code

	32	331	332	333	334	335	336	337	339	TOTAL
a. 1 to 4 Employees			1							1
b. 5 to 9 Employees	1		1	1	2					5
c. 10 to 19 Employees			1	3	3					7
d. 20 to 49 Employees	3		5	3	4		1		1	17
e. 50 to 99 Employees			3	4	2	1	3		1	14
f. 100 to 249 Employees	2		8	1	1	2	2		1	17
g. 250 to 499 Employees	1		1			1	2		1	6
h. 500 to 999 Employees			1							1
i. 1000 or more Employees					1		1			2
Blank				1						1

TOTAL	7	0	21	13	13	4	9	0	4	71

Source: Douglas Thomas and Brian Weiss. “Economics of Manufacturing Machinery Maintenance: A Survey and Analysis of U.S. Costs and Benefits.” NIST Advanced Manufacturing Series 100–34. https://doi.org/10.6028/NIST.AMS.100–34

**Table 3.** Stratifications

	Strata 1	Strata 2	Strata 3
	
Group 1	Industries: NAICS 321–333, 337 and Establishment size: 1 to 99 employees	1 to 19 employees	NAICS 321–332, 337
Group 2	Industries: NAICS 321–333, 337 and Establishment size: 100 or more employees	100 or more employees	NAICS 335–336, 339
Group 3	Industries: NAICS 334–336, 339 and Establishment size: 1 to 99 employees	20 to 99 employees	NAICS 333–334
Group 4	Industries: NAICS 334–336, 339 and Establishment size: 100 or more employees		
	

**Table 4.** Costs and Losses Associated with Maintenance –Estimated with no Stratification and no Monte Carlo analysis from Thomas and Weiss (2020)

	Estimate ($2016 Billion)	90 % Confidence Interval	
Costs	74.5	49.8	98.8
Direct Maintenance Costs	57.3	42.4	72.2
Costs due to Faults and Failures	16.3	7.1	25.5
Inventory Costs	0.9	0.3	1.1

Losses	119.1	43.9	197.3
Unplanned Downtime	18.1	10.4	27.8
*Labor*	*13.5*	*7.1*	*22.1*
*Capital Depreciation Buildings*	*2.5*	*1.8*	*3.1*
*Capital Depreciation Machinery*	*1.0*	*0.7*	*1.2*
*Energy*	*1.1*	*0.8*	*1.4*
Defects	0.8	0.0	2.7
Lost Sales	100.2	33.5	166.8
*Due to Defects*	*31.2*	*3.6*	*58.7*
*Due to Delays*	*69.0*	*29.8*	*108.1*

Total Costs and Losses	193.6	93.6	296.2

Source: Thomas, Douglas and Brian Weiss. “Economics of Manufacturing Machinery Maintenance: A Survey and Analysis of U.S. Costs and Benefits.” NIST Advanced Manufacturing Series 100–34. https://doi.org/10.6028/NIST.AMS.100-34

**Table 5.** Summary of Monte Carlo Results using Stratification ($Billion 2016)

	Min	Max	Mean	Median	Standard Deviation
Costs					
Direct Maintenance Costs	36.7	205.4	81.6	74.4	28.1
Additional Costs due to Faults/Failures	2.7	25.7	15.7	15.8	3.3
Inventory Costs	0.2	1.2	0.8	0.9	0.1

Losses					
Downtime Costs	14.8	22.1	18.4	18.4	1.4
Lost Sales	32.6	214.0	105.0	102.1	23.6
Losses due to Defects	0.0	2.3	0.5	0.3	0.4

TOTAL	87.1	470.6	222.0	211.8	

**Table 6.** Comparison of those with the top 50 % in using Reactive Maintenance to those in the Bottom 50 % (i.e., Group 1 compared to Group 2 and Group 3 together), Monte Carlo Results

Variable	Top or Bottom 50 %	Min	Max	Mean	% Change: Top to Bottom (Mean)	Median	% Change: Top to Bottom (Median)	Standard Deviation
Maintenance Cost To Shipment Ratio	Top	0.009	0.021	0.016	81.7%	0.017	93.7%	0.00
Maintenance Cost To Shipment Ratio	Bottom	0.010	0.051	0.029		0.032		0.01
Costs due to Faults/Failures as a ratio to Shipments.	Top	0.001	0.009	0.006	−51.8%	0.006	−52.0%	0.00
Costs due to Faults/Failures as a ratio to Shipments.	Bottom	0.001	0.005	0.003		0.003		0.00
Percent of Planned Production time that is downtime	Top	5.38	13.17	10.38	−52.7%	10.44	−51.8%	1.27
Percent of Planned Production time that is downtime	Bottom	2.40	6.91	4.91		5.03		0.67
Defect Rate	Top	0.04	3.20	2.03	−78.5%	2.34	−80.7%	0.62
Defect Rate	Bottom	0.09	0.71	0.44		0.45		0.09
Percent of Sales lost due to Delays Related to Maint.	Top	1.34	4.67	3.30	−73.0%	3.36	−72.7%	0.50
Percent of Sales lost due to Delays Related to Maint.	Bottom	0.05	1.53	0.89		0.92		0.27
Percent of Sales lost due to Defects Related to Maint.	Top	0.09	1.94	1.28	−49.4%	1.39	−52.6%	0.33
Percent of Sales lost due to Defects Related to Maint.	Bottom	0.00	1.14	0.65		0.66		0.18
Percent Increase in Inventory due to Maint.	Top	1.30	6.81	4.64	−51.2%	4.69	−50.3%	0.78
Percent Increase in Inventory due to Maint.	Bottom	0.02	3.89	2.26		2.33		0.61
Percent Reactive Maintenance	Top	65.00	72.79	68.75	−67.7%	68.73	−67.6%	1.09
Percent Reactive Maintenance	Bottom	17.96	26.74	22.24		22.26		1.24

Items shown in RED indicate unexpected outcome in comparing the top and bottom

**Table 7:** Comparison of those with the top 50 % in using Predictive Maintenance to the Bottom 50 % (Group 2 compared to Group 3), Monte Carlo Results

Variable	Top or Bottom 50 %	Min	Max	Mean	% Change: Bottom to Top (Mean)	Median	% Change: Top to Bottom (Median)	Standard Deviation
Maintenance Cost To Shipment Ratio	Bottom	0.005	0.022	0.014	212.3%	0.014	237.1%	0.002
Maintenance Cost To Shipment Ratio	Top	0.008	0.076	0.043		0.048		0.014
Costs due to Faults/Failures as a ratio to Shipments.	Bottom	0.000	0.005	0.002	44.6%	0.002	40.3%	0.001
Costs due to Faults/Failures as a ratio to Shipments.	Top	0.000	0.006	0.003		0.003		0.001
Percent of Planned Production time that is downtime	Bottom	2.03	8.33	5.34	−18.5%	5.67	−22.5%	1.06
Percent of Planned Production time that is downtime	Top	1.37	7.00	4.35		4.39		0.77
Defect Rate	Bottom	0.15	1.31	0.75	−87.3%	0.77	−84.4%	0.17
Defect Rate	Top	0.01	0.19	0.09		0.12		0.05
Percent of Sales lost due to Delays Related to Maint.	Bottom	0.00	1.75	0.55	112.7%	0.64	96.0%	0.24
Percent of Sales lost due to Delays Related to Maint.	Top	0.00	2.00	1.17		1.25		0.46
Percent of Sales lost due to Defects Related to Maint.	Bottom	0.00	1.17	0.52	51.5%	0.60	36.7%	0.22
Percent of Sales lost due to Defects Related to Maint.	Top	0.00	1.57	0.79		0.82		0.28
Percent Increase in Inventory due to Maint.	Bottom	0.00	5.01	2.53	−22.5%	2.73	−19.3%	0.94
Percent Increase in Inventory due to Maint.	Top	0.00	3.89	1.96		2.20		0.79
Percent Predictive	Bottom	5.00	14.55	10.41	321.5%	10.42	324.0%	1.16
Percent Predictive	Top	31.70	54.90	43.88		44.17		3.50
Percent Reactive	Bottom	14.63	32.00	23.42	−10.6%	23.36	−10.5%	1.98
Percent Reactive	Top	16.10	25.10	20.93		20.92		1.56

Items shown in RED indicate unexpected outcome in comparing the top and bottom
